# Combined robot-assisted surgery of upper and lower genitourinary tract with excellent early functional and oncological outcome – a case report of a concomitant nerve-sparing radical prostatectomy, diverticulectomy and ureterectomy

**DOI:** 10.1186/s12894-025-01901-9

**Published:** 2025-09-02

**Authors:** Jan Lüdecke, Mirjam Naomi Mohr, Till Rasmus Schneider, Joost Wilhelm Voß, Arne Strauß, Lutz Trojan, Mathias Reichert

**Affiliations:** Department of Urology, University Medical Center Goettingen, Robert-Koch-Str. 40, Goettingen, 37075 Germany

**Keywords:** Prostate cancer, CAKUT, Nerve-sparing radical prostatectomy, Robot-assisted surgery, NeuroSAFE, Case report

## Abstract

**Introduction and importance:**

"Congenital anomalies of the kidney and urinary tract" (CAKUT) represent a heterogeneous and rare group of disorders affecting the urinary tract. Despite the widespread availability of ultrasound, the diagnosis of congenital urinary tract malformations is often made in adulthood. As a result, CAKUT are typically incidental findings during imaging investigations. This case report represents the first description of a simultaneous surgical therapy of CAKUT and nerve-sparing-prostatectomy, demonstrating that concomitant surgery does not present disadvantages and that a successful nerve-sparing radical prostatectomy can still be performed under NeuroSAFE control.

**Case presentation:**

In this report, we describe a patient with prostate adenocarcinoma who had an incidental finding of left-sided CAKUT, including kidney aplasia, ureterocele, and bladder diverticulum. The patient underwent a simultaneous nerve-sparing radical prostatectomy, ureterectomy, and diverticulectomy.

After catheter removal on day 10 after surgery, the patient demonstrated adequate continence (6gr in 24 h pad test, 450 ml voiding volume, no residual urine). The pathological examination revealed a pT2c pN0 (0/5) R0, GS 3 + 4 = 7a adenocarcinoma of the prostate.

The patient expressed high satisfaction with the surgery and its outcomes.

**Conclusion:**

This case demonstrates for the first time that a combined approach to complex surgeries of both the upper and lower genitourinary tracts, addressing oncological considerations, can be performed safely without compromising early functional or early oncological outcomes.

## Introduction

Unilateral renal aplasia is more prevalent in males than in females and is more frequently found on the left side [1]. This condition is classified as a type of CAKUT (congenital anomalies of the kidney and urinary tract) and may be associated with other anomalies, such as ureterocele, with a worldwide incidence of approximately 4–60 of 10000 births and are mostly diagnosed during pre- and postnatal phase [2, 3]. The most common symptom of CAKUT is symptomatic urinary tract infections. However, symptoms are not always present. In asymptomatic cases, most CAKUT conditions are incidental findings in adults [4]. Treatment is not necessary if there are no symptoms. While surgical interventions can improve symptom control, they do not necessarily enhance renal function.

In this report, we describe a patient with prostate adenocarcinoma who had an incidental finding of left-sided CAKUT, including kidney aplasia, ureterocele, and bladder diverticulum. The patient underwent a simultaneous nerve-sparing radical prostatectomy, ureterectomy, and diverticulectomy. This case report represents the first description of this surgical approach, demonstrating that concomitant surgery does not present disadvantages and that a nerve-sparing radical prostatectomy can still be performed under NeuroSAFE control.

## Case report

### Clinical summary

A 41-year-old male patient was referred to our urology department for evaluation of prostate adenocarcinoma. Even though histopathological analysis revealed a Gleason score of 3 + 3 = 6, with two positive biopsy cores from the left side (2 out of 6) and one positive core from the right lobe (1 out of 6), the patient whished for active treatment and refused Active-Surveillance. The patients initial prostate-specific antigen (PSA) level was measured at 10.32 ng/ml. Notably, the digital rectal examination did not reveal any suspicious findings in the prostate.

### Diagnostics

The patient underwent a computed tomography (CT) scan for staging purposes, which revealed localized prostate carcinoma without evidence of metastasis. Notably, the CT scan also identified incidental findings, including a left-sided megaureter associated with a ureterocele and a complete absence of the kidney (kidney aplasia). Multiple calculi were detected within the megaureter, which extended to the left iliac artery and vein.

**Fig. 1:**
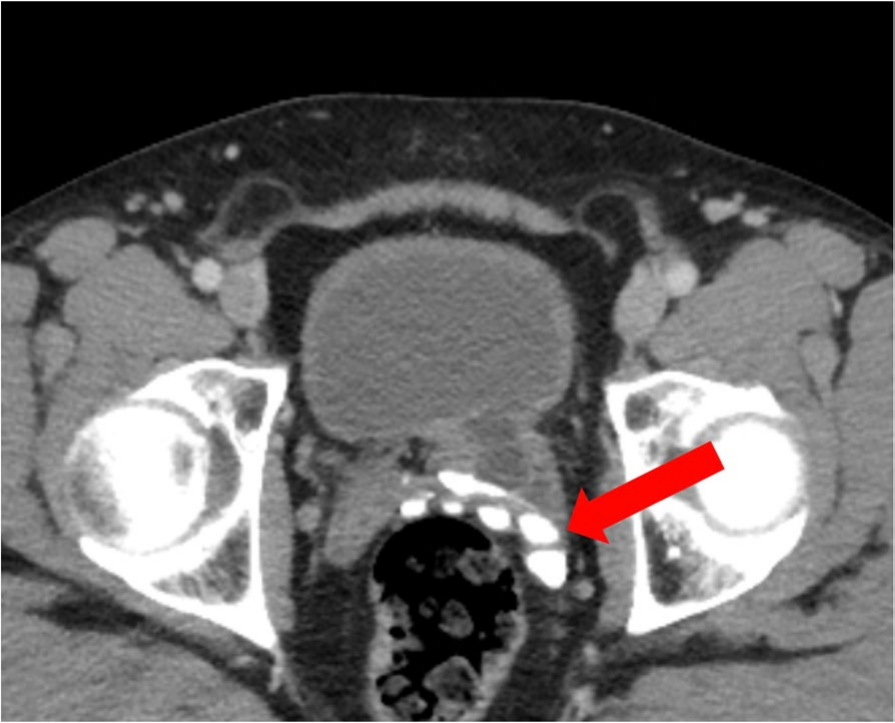
Computed tomography, transverse view demonstrating calcification of the left seminal vesicle

In addition to the stones in the megaureter, significant calcification of the left seminal vesicle was observed (see Fig. 1).

Due to the findings from the computed tomography, we performed a cystoscopy to assess the anatomical situation of the bladder neck and the ureteral orifices on both sides. During the cystoscopy, the ureterocele was found to be located in a diverticulum, and the orifice of the ureter could not be identified, either during cystoscopy or via -graphy.

Due to the patient's young age and his will, a robot-assisted nerve-sparing radical prostatectomy was planned for the curative treatment of the adenocarcinoma of the prostate. Additionally, a simultaneous resection of the entire left ureter, along with the diverticulum and ureterocele, was planned, since it was believed, that without resection, the functional outcome would have been compromised. However, a compelling indication for resection because of recurrent febrile urinary tract infection (fUTI) was not given.

### Surgery

**Fig. 2:**
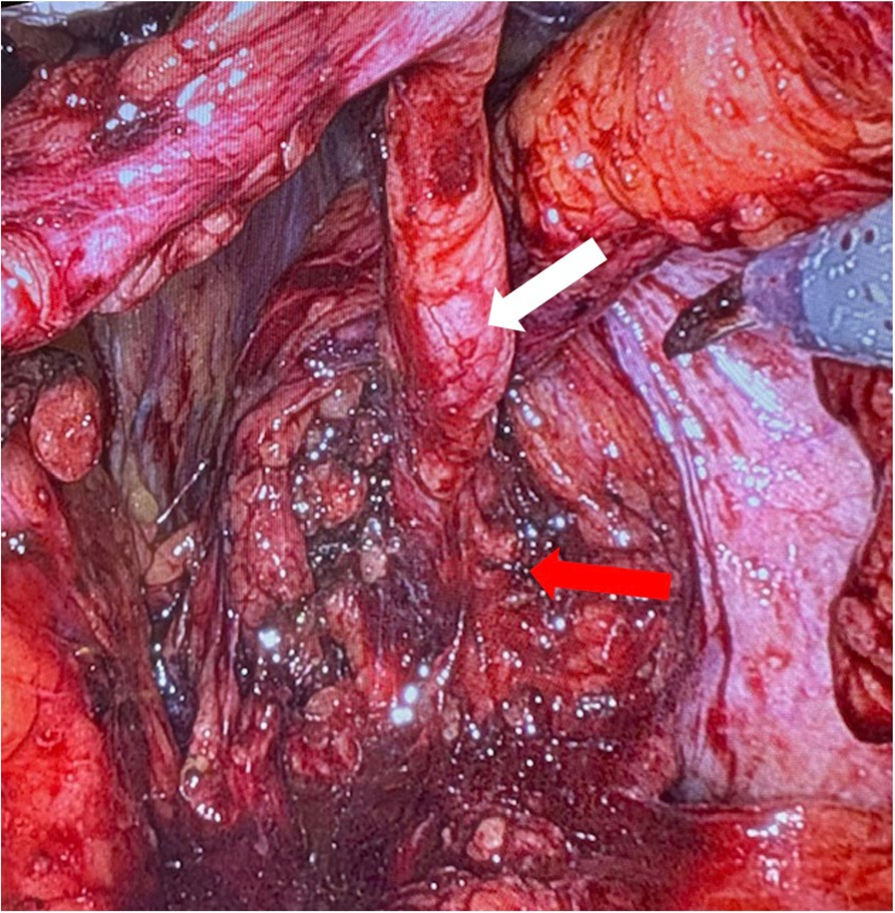
Mobilized left ureter (white arrow) extending to the bladder wall (red arrow)

For the prostatectomy, we routinely use a descending approach, utilizing NeuroSAFE [5] for the nerve-sparing procedure. The first step involved mobilizing the left ureter. To achieve this, we incised the peritoneum on the lateral side of the left ligamentum umbilicale mediale. The urachus, the medial umbilical ligaments on both sides, and the median umbilical ligament remained attached to the umbilicus. After identifying the left ureter, we followed it to the bladder wall on the left side (see Fig. 2) by resecting the left ductus deferens, the ligamentum umbilicale (near the A. iliaca interna), and the A. vesicalis superior.

**Fig. 3:**
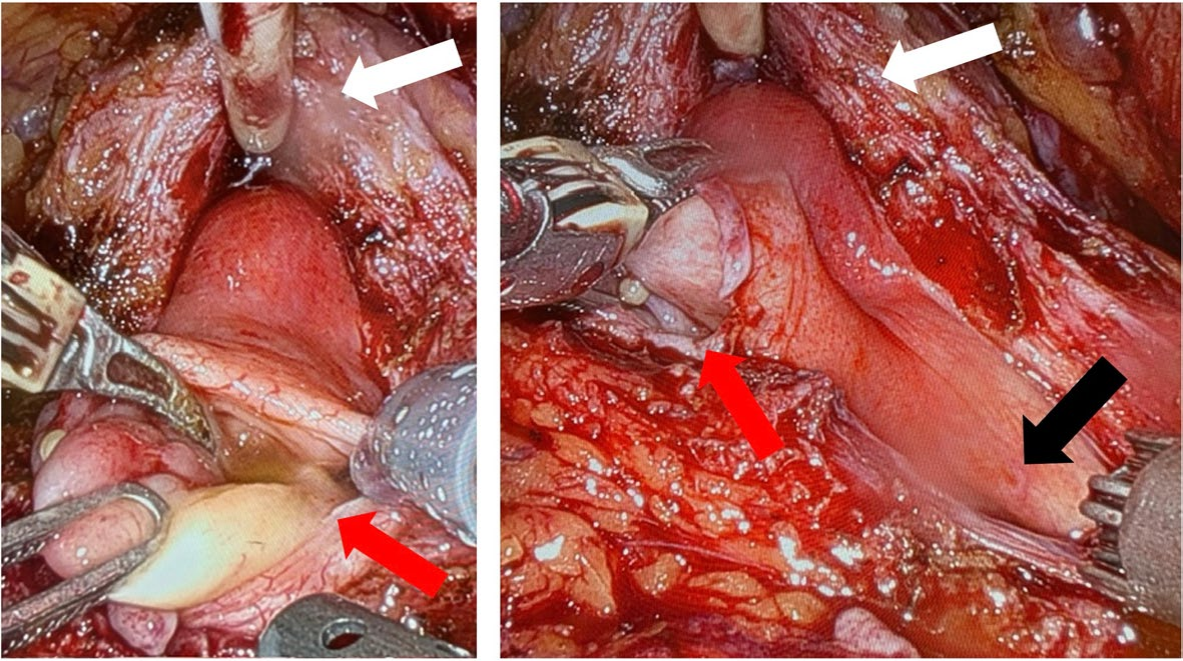
Ventral view of the opened bladder neck (white arrow), showing the diverticulum on the left side (red arrow) and its positional relationship to the adjacent structures on the right (black arrow = right ureteral orifice)

After mobilizing the bladder, a ventral incision was made in the bladder neck between the bladder and the prostate until the inserted catheter was visualized. Prior to dissecting the dorsal bladder neck, the diverticulum on the left trigonal side was identified (see Fig. 3).

Despite the findings during cystoscopy and cystography, the diverticulum extended significantly behind the prostate and seminal vesicles, proving to be much larger than anticipated. The left ureteral orifice was not patent and was covered by a thin layer of urothelium. A considerable amount of sludge was found within the diverticulum, which had been identified as calcifications in the preoperative computed tomography.

**Fig. 4:**
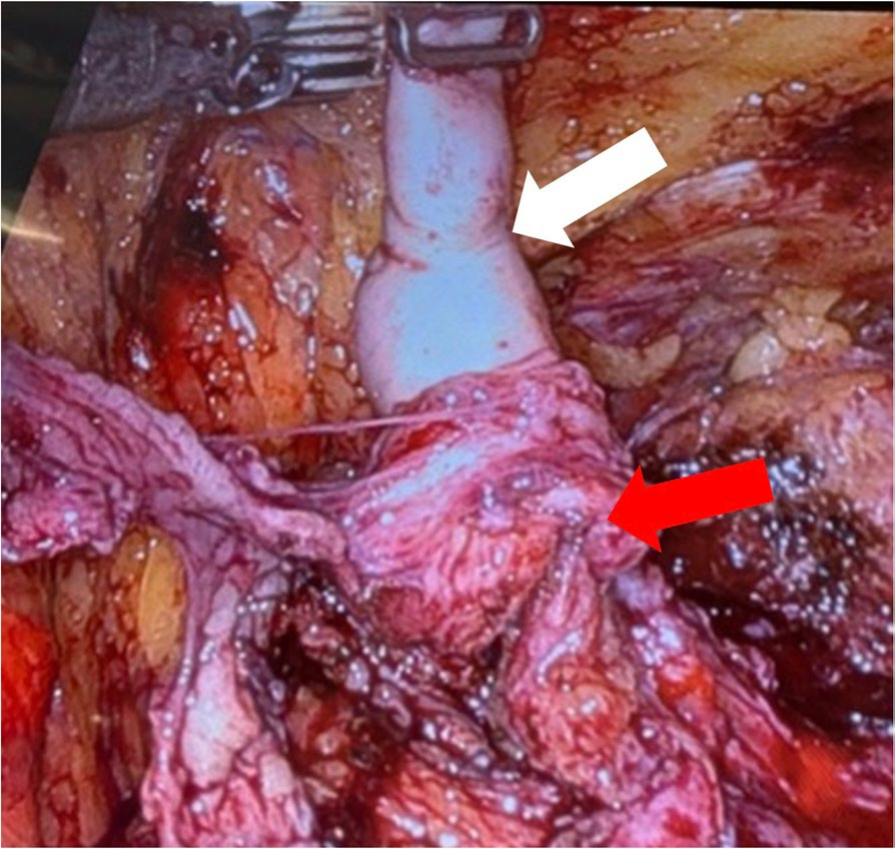
Stripped left ureter (white arrow), remaining adherent to the bladder wall (red arrow)

Due to the risk of damaging the rectum located posterior to the diverticulum, which was deeply adherent behind the prostate, we opted not to mobilize or dissect the diverticulum from the opened bladder. Instead, we inserted a urethral catheter into the left ureter and performed a stripping procedure (see Fig. 4).

The dorsal bladder neck was incised, allowing for the dissection of the seminal vesicles and ducts. The distal end of the diverticulum was subsequently isolated from behind the prostate. Following the stripping of the ureter and dissection of the diverticulum wall, it became possible to resect the entire left ureter along with the diverticulum.

A standardized radical prostatectomy was then performed using an intraoperative frozen section specimen, which facilitated a successful nerve-sparing procedure on both sides. Due to the partial bladder resection, it was necessary to create a new bladder neck. A running suture was initiated at the far left side of the opened bladder, extending toward the original dorsal bladder neck for the prostatectomy. Ultimately, a bladder neck was formed that allowed for a vesicourethral running anastomosis, as described by Van Velthofen et al. [6]. Following the completion of the reconstructive phase, a bilateral pelvic lymphadenectomy was performed. The duration of the entire procedure was 268 min.

### Postoperative course

**Fig. 5:**
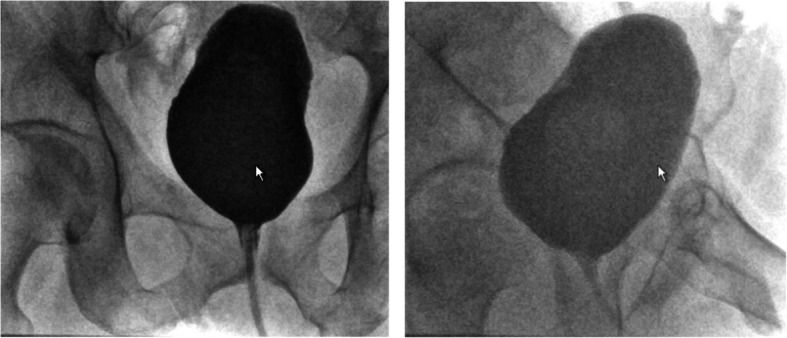
Postoperative cystography (anterior–posterior and lateral right) performed 10 days after surgery

The indwelling catheter was retained for 10 days due to the reconstruction of the bladder neck. The patient was initially discharged from the hospital after 4 days of an uncomplicated recovery. Ten days post-surgery, a cystography was performed, which revealed a tight anastomosis (Fig. 5).

After catheter removal, the patient demonstrated adequate continence (6gr in 24 h pad test, 450 ml voiding volume, no residual urine). The pathological examination revealed a pT2c pN0 (0/5) R0, GS 3 + 4 = 7b adenocarcinoma of the prostate. The ureter measured 9.1 cm in length and exhibited no pathological features, nor did the diverticulum.

The patient expressed high satisfaction with the surgery and its outcomes.

## Discussion

Robot-assisted surgery has become the standard in the field of intra-abdominal uro-oncology and reconstructive surgery [7]. Following the initial presentation of robot-assisted laparoscopic prostatectomy (RARP) by Binder et al. [8], RARP is now considered the standard of care. Khalil et al. presented a case report in which they performed a prostatectomy simultaneously with a ureterectomy and a heminephrectomy [9]. In this case, they were compelled to resect both the prostate and the anomaly of the upper urinary tract due to an ectopic ureter insertion into the prostatic urethra. Given the anomalous insertion of the ureter into the prostate, along with the presence of advanced localized prostate cancer, they were unable to perform a nerve-sparing procedure. A single-port approach was utilized, and this type of procedure was reported for the first time.

By now, it is not uncommon to address both, upper and lower urinary tract, at the same time, for example when two (or more) malignancies are present by the time of surgery [10]. A case series by Pisipati et al. demonstrated that in carefully selected patients a combined robotic surgery can reduce morbidity and complications [10]. Scarcella et al. come to the same conclusion in their review, in which they address all published reports of combined upper and lower urinary tract surgeries [11].

In our case, we encountered a low-risk prostate carcinoma that was suitable for nerve preservation. Fortunately, the patient underwent a computed tomography (CT) scan for staging purposes, which revealed a congenital anomaly of the kidney and urinary tract (CAKUT), including kidney aplasia (complete absence of the kidney), ureterocele, and a diverticulum on the left side. The left ureter was traceable from the bladder to just above the left iliac artery and vein.

This is the first documented case in which all three conditions—adenocarcinoma of the prostate, malformation of the bladder and CAKUT affecting the left upper tract—were addressed simultaneously.

Given that our patient did not exhibit any symptoms related to the diverticulum and the left-sided ureterocele associated with the solitary left ureter, it is worth considering whether these conditions should be addressed concurrently with the prostatectomy in a single surgical procedure. In this instance, we appeared to be compelled to perform a concomitant procedure for both issues, as the ureter could not be left in situ due to calcification and the associated risk of infection. Since the left kidney was completely absent, there was no reason, like preserving renal function, etc., for leaving the remaining anatomical structures of the left-sided upper urinary tract in situ.

As mentioned in the surgery part of the manuscript, no calcificated stones but only sludge was identified in the diverticulum intraoperatively, which was automatically sucked away and therefore no material was available for stone composition analysis. Since no fUTIs were present in the patient’s medical history, the circumstance of having no material to examine seems not to be crucial and there seems to be no higher risk of infections. It can be assumed that the sludge was caused by the lack of diuresis on the left side or the stagnant urine in the diverticulum, respectively.

In our opinion, a staged procedure would have been risky. Intraoperatively, the diverticulum was found to be significantly larger than what was indicated by the CT scan and cystoscopy, extending behind the prostate and caudal to the left seminal vesicle. Performing solely the prostatectomy trying to leave the diverticulum in situ would have led to accidental openings of the diverticulum. In addition to that, without resection of the diverticulum-neck the vesicourethral anastomosis could not be performed.

In addition to the intraoperative conditions, that almost “forced” a concomitant surgery, the benefit for the patient speaks for itself.

In contrast to the case presented by Khalil et al., which involved a combined surgery of the upper urinary tract and a prostatectomy [9], we undertook a complex reconstruction of the urinary bladder while ensuring the preservation of functional capabilities. Despite the resection of the diverticulum, successful bilateral nerve preservation was achieved. We standardly perform nerve preservation under NeuroSAFE control [5].

This case demonstrates for the first time that a combined approach to complex surgeries of both the upper and lower genitourinary tracts, addressing oncological considerations, can be performed safely without compromising functional or oncological outcomes. Following catheter removal, the patient exhibited adequate voiding capabilities, and early continence was more than satisfactory.

## Conclusion

This case demonstrates for the first time that a combined approach to complex surgeries of both the upper and lower genitourinary tracts, addressing oncological considerations, can be performed safely without compromising early functional or early oncological outcomes. Following catheter removal, the patient exhibited adequate voiding capabilities, and early continence was more than satisfactory.

Of course, long-term outcome has to be followed.

### Declaration of generative AI and AI-assisted technologies in the writing process

During the preparation of this work the author(s) used “GPT-4o mini” (OpenAI) in order to improve english language. After using this tool, the author(s) reviewed and edited the content as needed and take(s) full responsibility for the content of the publication.

## Data Availability

No datasets were generated or analysed during the current study.
